# Dosimetric Discrepancy Between Whole-Lung and Lobe-Specific Metrics in Lung Stereotactic Body Radiotherapy and Its Implications for Regional Dose Assessment

**DOI:** 10.7759/cureus.102180

**Published:** 2026-01-23

**Authors:** Hideharu Miura, Shuichi Ozawa, Masahiro Kenjo

**Affiliations:** 1 Department of Radiation Oncology, Hiroshima High-Precision Radiotherapy Cancer Center, Hiroshima, JPN; 2 Department of Radiation Oncology, Institute of Biomedical and Health Sciences, Hiroshima University, Hiroshima, JPN

**Keywords:** auto-segmentation, dose-volume histogram, lobe-based dosimetry, lung tumor, stereotactic body radiotherapy

## Abstract

Introduction: Stereotactic body radiotherapy (SBRT) is a standard treatment for early-stage non-small cell lung cancer (NSCLC). Radiation pneumonitis is a major dose-limiting toxicity, typically assessed using whole-lung metrics like mean lung dose (MLD) and relative lung volume receiving at least 20 Gy (V_20Gy_). However, the high-dose conformality of SBRT may lead to a volumetric averaging effect, where whole-lung metrics obscure intense local doses within a small fraction of the lung. This study quantitatively evaluates the discrepancy between conventional whole-lung metrics and lobe-specific dosimetry.

Methods: We retrospectively analyzed 10 patients with T1-T2N0M0 NSCLC treated with SBRT (42 Gy in four fractions). Lung lobes - right upper, middle, lower, and left upper and lower - were automatically segmented using deep learning-based software (OncoStudio; Oncosoft, Seoul, South Korea) and manually refined. The lobe containing the tumor was defined as the tumor-bearing lobe (TBL), while others were defined as non-tumor-bearing lobes (NTBLs). Dose-volume histogram (DVH) parameters (MLD, relative lung volume receiving at least 5 Gy (V_5Gy_), relative lung volume receiving at least 10 Gy (V_10Gy_), and V_20Gy_) were compared for the whole lungs, TBL, and NTBLs using the Wilcoxon signed-rank test. Spearman’s rank correlation coefficient assessed the relationship between whole-lung and lobe-specific metrics.

Results: TBL-specific dose intensities were markedly higher than whole-lung metrics: median V_20Gy_ was 6.3% for TBL versus 1.5% for the whole lungs, and median MLD was 4.61 Gy for TBL versus 1.68 Gy for the whole lungs. Statistically significant differences were observed across all parameters (*p* < 0.01). Correlation analysis showed that statistically significant correlations were not reached between whole-lung metrics and TBL values, including for V_20Gy_ (*p* = 0.074). Instead, these whole-lung metrics exhibited strong and consistent correlations with the dose distributed across the combined NTBLs.

Conclusion: Conventional whole-lung metrics substantially underestimate the radiation concentration within the TBL due to volumetric dilution. While high-dose whole-lung parameters like V_20Gy_ reflect local lobar intensity, low-dose metrics primarily serve as surrogates for NTBL exposure. Lobe-based analysis provides a refined, anatomically grounded framework for characterizing regional dose distributions in lung SBRT and reveals spatial dose patterns that are obscured when using conventional whole-lung assessment alone.

## Introduction

Stereotactic body radiotherapy (SBRT) is an established treatment option for early-stage non-small cell lung cancer (NSCLC), particularly for patients who are medically inoperable or prefer a non-invasive approach [1-3]. Despite its clinical efficacy, radiation pneumonitis is a primary dose-limiting toxicity [4-8]. Traditional radiation pneumonitis risk assessment focuses on whole-lung metrics, such as the mean lung dose (MLD) and relative lung volume receiving at least 20 Gy (V_20Gy_), derived from the total bilateral lung volume [4-8]. However, these whole-lung parameters may lack the sensitivity required for SBRT due to its highly conformal dose distribution and steep dose gradients. Because the high-dose region is concentrated within a small fraction of the parenchyma, whole-lung metrics are inherently susceptible to a volumetric averaging effect [4]. In larger lungs, this effect can obscure intense local doses, potentially leading to an underestimation of regional toxicity and reducing the performance of dose-response models.

Instead of relying solely on whole-lung metrics, analyzing dose distributions on a lobe-by-lobe basis offers a more detailed understanding of regional lung damage [9]. Although anatomical lobe-specific dosimetry has long been recognized for its clinical potential, its routine application was historically hindered by the labor-intensive nature of manual contouring. Recently, artificial intelligence (AI)-based auto-segmentation has overcome this limitation by enabling the rapid and accurate delineation of individual lung lobes on standard planning CT images [10-13]. This technological shift allows for detailed regional dosimetric assessment within existing clinical workflows. Despite these advancements, the correlation between lobe-specific dosimetric parameters and standard whole-lung metrics has not yet been thoroughly established.

This study aimed to quantitatively evaluate the discrepancy between conventional whole-lung dose-volume metrics and lobe-specific dosimetry in lung SBRT. This dosimetric and analytic study focused on the methodological feasibility of lobe-specific evaluation; notably, clinical outcomes such as radiation pneumonitis were not included in the analysis. By establishing this lobe-based dosimetric framework, we sought to provide a more anatomically precise description of regional lung dose than is possible with conventional whole-lung assessment.

## Materials and methods

Patient characteristics

A total of 10 consecutive patients with early-stage NSCLC were enrolled in this retrospective study. All patients were treated with SBRT at the Hiroshima High-Precision Radiotherapy Cancer Center, Hiroshima, Japan, between November 2023 and August 2024. This study was approved by the Hiroshima University Institutional Review Board (approval no. E2017‑0947‑02). Inclusion criteria required a complete planning CT dataset and a definitive diagnosis of T1-T2N0M0 NSCLC.

CT simulation and treatment planning

All patients underwent planning CT simulations using a GE Optima 580 scanner (GE Healthcare, Waukesha, USA), with a slice thickness of 1.25 mm. The respiratory management strategy was individualized for each patient based on the magnitude of tumor motion observed during pre-simulation fluoroscopy or four-dimensional CT (4DCT). For cases with a small respiratory excursion, scans were acquired under free-breathing conditions to define the internal target volume (ITV). For patients with significant tumor motion, a voluntary end-expiratory breath-hold technique was utilized to stabilize the target, as free-breathing acquisition would have resulted in excessively large target volumes. The gross tumor volume (GTV) was manually delineated on the planning CT images by an experienced radiation oncologist. The planning target volume (PTV) was then generated by applying a 5-mm margin to the ITV according to our institutional protocol.

Treatment plans were developed in the Eclipse treatment planning system (Varian Medical Systems, Palo Alto, USA) using 6-MV or 10-MV flattening filter-free (FFF) beams. All plans utilized volumetric modulated arc therapy (VMAT) with partial arcs directed from the ipsilateral side to minimize the dose to the contralateral lung. To ensure high-resolution accuracy for SBRT dosimetry, dose distributions were calculated using the Acuros XB algorithm with a 1.0-mm grid size. The standard prescription dose was 42 Gy in four fractions, with the plan normalized to cover 95% of the PTV while strictly adhering to institutional organ-at-risk (OAR) constraints. Key lung constraints included a relative lung volume receiving at least 15 Gy (V_15Gy_) of ≤ 25%, V_20Gy_ of ≤ 20%, and a mean dose of ≤ 18 Gy. The spinal cord was limited to a maximum dose of 25 Gy.

AI-based lobe segmentation and classification

For the dosimetric analysis, "whole lungs" were defined as the bilateral pulmonary parenchyma excluding the GTV. The trachea and proximal bronchial tree were also excluded from the lung volumes to ensure dosimetric consistency. This definition was applied uniformly across all 10 patients. The individual lung lobes - right upper (RUL), right middle (RML), right lower (RLL), left upper (LUL), and left lower (LLL) - were automatically segmented on the planning CT images using OncoStudio (Oncosoft, Seoul, South Korea), a deep learning-based auto-segmentation software. To ensure anatomical accuracy, a medical physicist with over 15 years of experience manually reviewed AI-generated contours. Manual refinements were primarily performed to correct automatic delineations where lobe overlaps occurred along the fissures and at the boundaries near the mediastinum and diaphragm. The average manual editing time was approximately five minutes per case. For each patient, the lobe containing the GTV was identified as the tumor-bearing lobe (TBL). The remaining four lobes were evaluated collectively as the non-tumor-bearing lobes (NTBLs) to assess the dose distribution between the involved and uninvolved pulmonary tissue.

Dosimetric evaluation

Dose-volume histogram (DVH) parameters were calculated for the whole lungs, TBL, and NTBLs using the treatment planning system. The primary metrics included MLD and relative lung volume receiving at least 5 Gy (V_5Gy_), relative lung volume receiving at least 10 Gy (V_10Gy_), and V_20Gy_. Statistical analyses were conducted to characterize the distribution and relationship between whole-lung and lobe-specific metrics.

Statistical analysis

Continuous variables were expressed as median values with interquartile ranges (IQRs). To evaluate the discrepancy between whole-lung and lobe-specific dose distributions, paired comparisons of dosimetric metrics (MLD, V_5Gy_, V_10Gy_, and V_20Gy_) among the TBL, NTBLs, and whole lungs were performed using the Wilcoxon signed-rank test. The relationship between global and regional dosimetry was assessed using Spearman’s rank correlation coefficient (*ρ*). Statistical significance was defined as *p* < 0.05 for all tests. Data visualization was performed using Python (version 3.11; Python Software Foundation, Wilmington, USA), employing the Matplotlib and SciPy libraries. Statistical computations were conducted using R (version 4.4; R Foundation for Statistical Computing, Vienna, Austria). Due to the exploratory nature of this study and the small sample size, no formal correction for multiple testing was applied. The *p*-values should be interpreted as descriptive indicators of potential differences rather than definitive confirmatory results.

## Results

Demographic data

The cohort had a median age of 81 years (range: 66-90 years), with a male predominance (80%). Most tumors were located in the lower lobes (70%), as summarized in Table 1. The study focused on relatively small lesions, with a median GTV of 2.2 cm^3^ and PTV of 14.1 cm^3^. These characteristics reflect a typical clinical population undergoing SBRT for early-stage peripheral lung tumors.

**Table 1:** Patient and tumor characteristics (n = 10) RUL: right upper lobe; RML: right middle lobe; RLL: right lower lobe; LUL: left upper lobe; LLL: left lower lobe; IQR: interquartile range; GTV: gross tumor volume; PTV: planning target volume

Parameter	Value
Age (years)	
Median (range)	81 (66 - 90)
Gender	
Male, n (%)	8 (80.0%)
Female, n (%)	2 (20.0%)
Tumor location (lobe)	
RUL, n (%)	1 (10.0%)
RML, n (%)	0 (0%)
RLL, n (%)	6 (60.0%)
LUL, n (%)	2 (20.0%)
LLL, n (%)	1 (10.0%)
Tumor volume (cm^3^)	
GTV, median (IQR)	2.2 (0.6-3.0)
PTV, median (IQR)	14.1 (10.1-19.1)

Volumetric analysis of lung subunits

**Figure 1:**
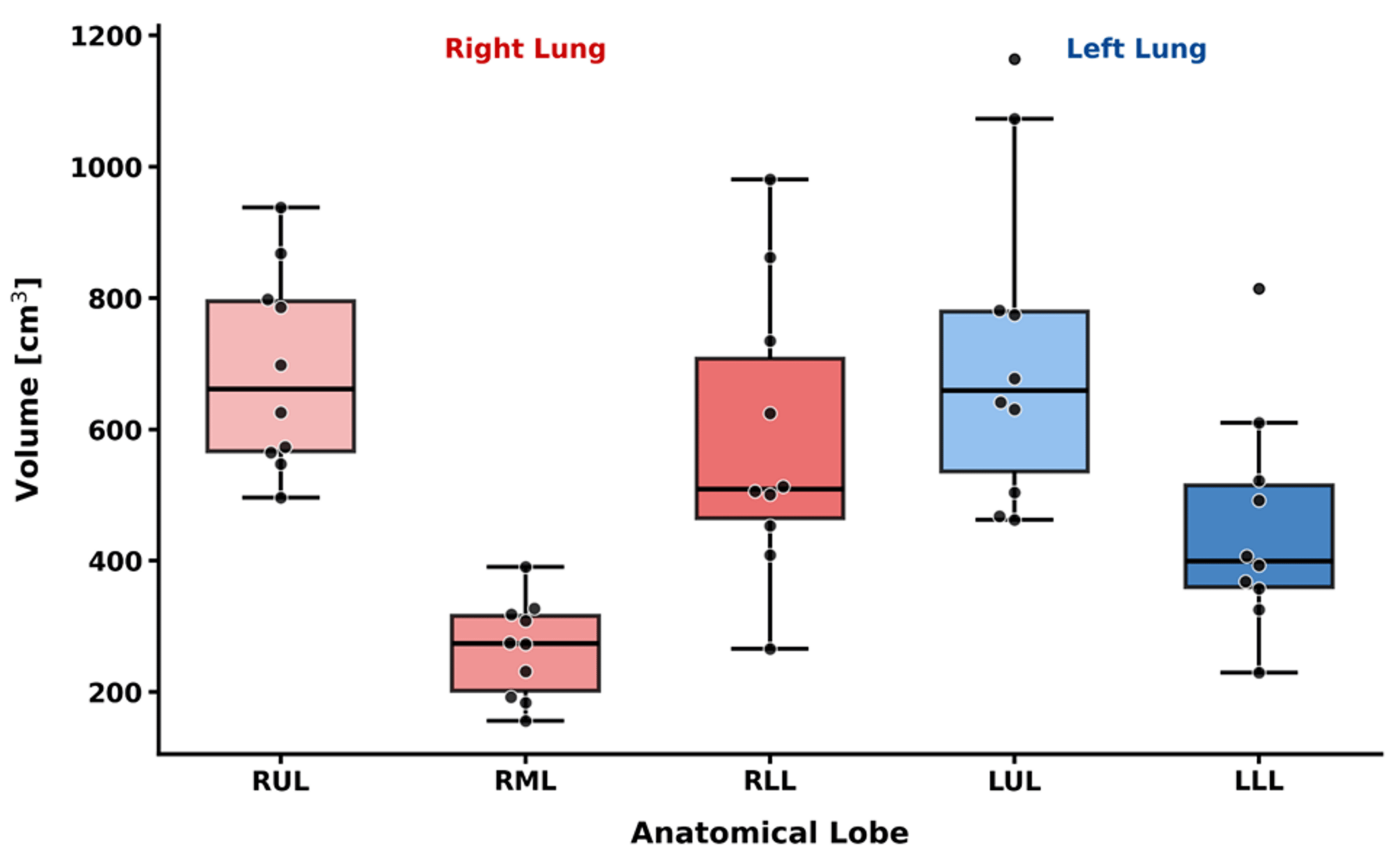
Volumetric distribution of individual lung lobes Box-and-whisker plots representing the volumetric distribution of the five lung lobes across the patient cohort (n = 10). Individual data points for each patient are overlaid on the plots. The horizontal line within each box indicates the median value, while the whiskers extend to the minimum and maximum values within 1.5 times the interquartile range; points beyond the whiskers represent outliers. RUL: right upper lobe; RML: right middle lobe; RLL: right lower lobe; LUL: left upper lobe; LLL: left lower lobe

Figure 1 illustrates the volumetric distribution of the five individual lung lobes. The median volumes (IQRs) were as follows: RUL 662.0 cm^3^ (566.7-795.2 cm^3^); RML 273.9 cm^3^ (201.8-315.8 cm^3^); and RLL 509.3 cm^3 ^(465.0-707.4 cm^3^). For the left lung, the median volumes (IQRs) were LUL 659.2 cm^3^ (535.8-779.3 cm^3^) and LLL 399.9 cm^3^ (360.1-514.7 cm^3^). The median total lung volume (TLV) was 2504.4 cm^3^ (IQR: 2129.4-3112.4 cm^3^).

Dosimetric discrepancy between whole-lung and lobe-specific metrics

**Figure 2:**
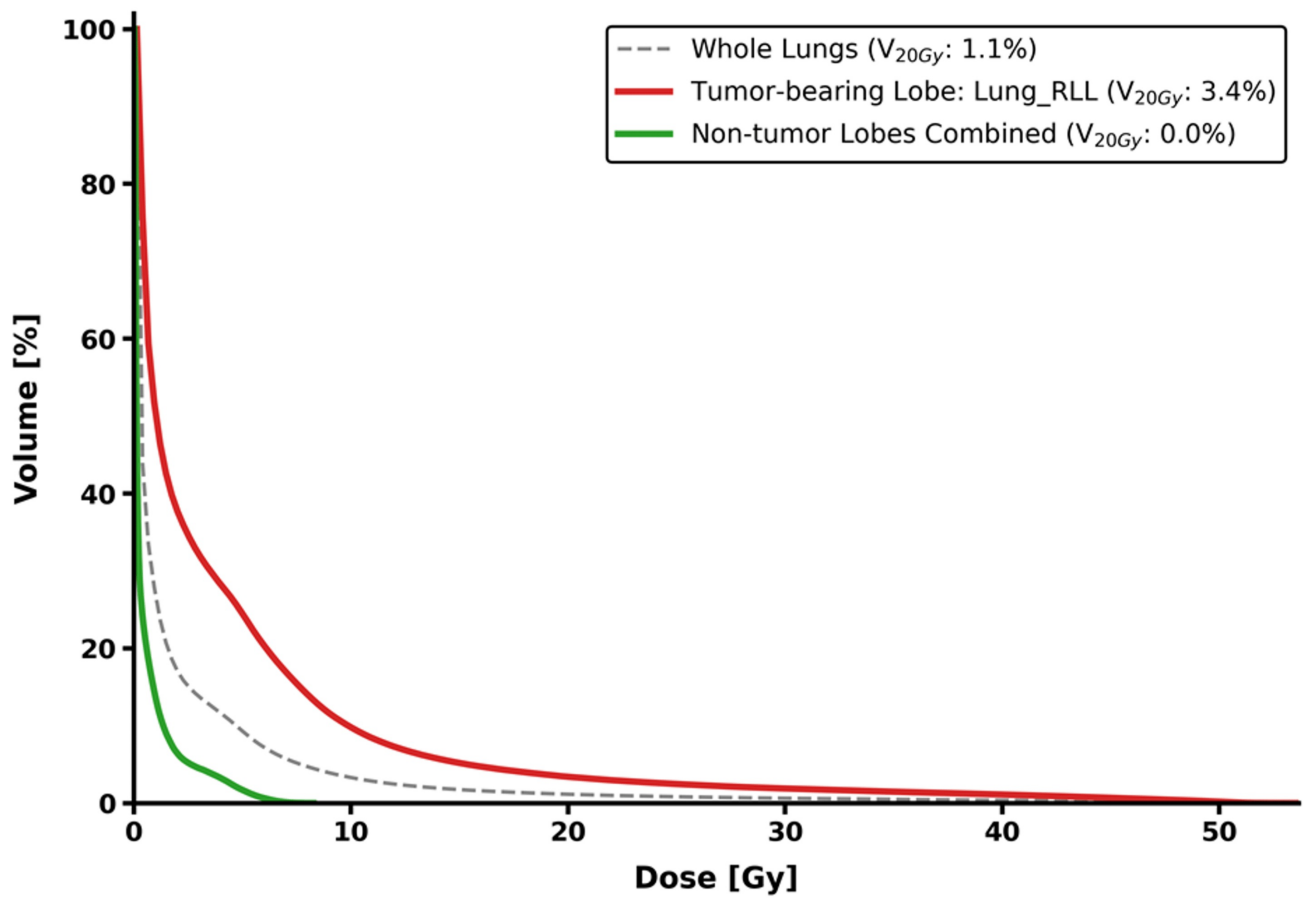
Comparison of dose-volume histogram (DVH) curves by anatomical subunit Representative cumulative dose-volume histograms for a single patient, comparing the whole lungs (dashed line), the TBL (red solid line), and the combined NTBLs (green solid line). The TBL exhibits a substantially higher dose profile (V_20Gy_ = 3.4%) compared to the whole-lung metric (V_20Gy_ = 1.1%), whereas the NTBLs receive negligible high-dose exposure (V_20Gy_ = 0.0%). RLL: right lower lobe; TBL: tumor-bearing lobe; NTBLs: non-tumor-bearing lobes; V_20Gy_: relative lung volume receiving at least 20 Gy

Figure 2 illustrates the discrepancy between whole-lung and lobe-specific dose assessments on DVH curves. While the whole-lung assessment suggested a diluted dose distribution, lobe-specific analysis revealed a marked localization of high-dose volumes within the TBL. For instance, the V_20Gy_ was substantially higher in the TBL compared to the whole lungs. In contrast, while the NTBLs remained affected by low-dose spillage, their contribution to the high-dose volume was negligible.

Comparative dosimetry across lung subunits

**Figure 3:**
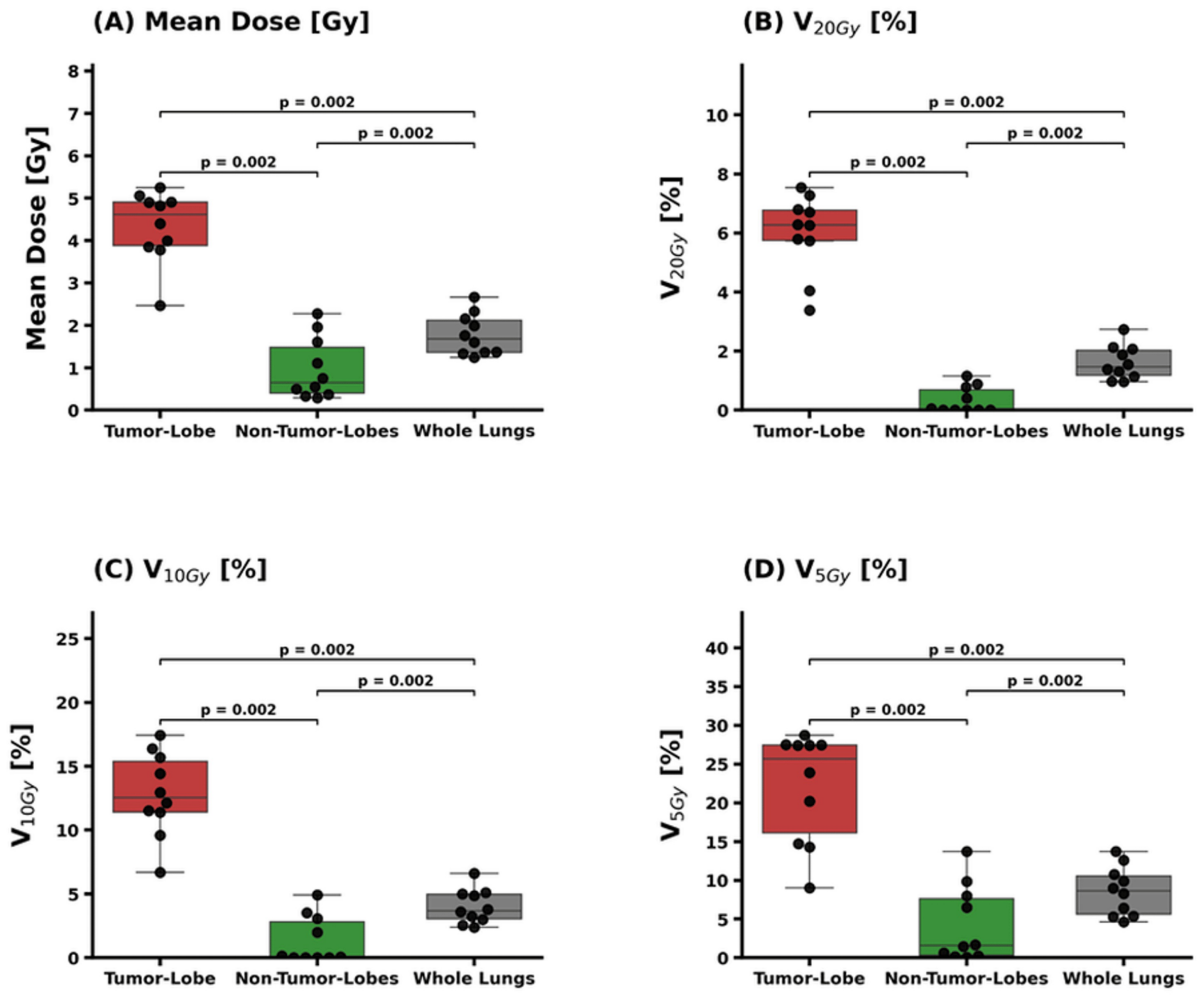
Quantitative comparison of dosimetric parameters between lung subunits Comparison of (A) MLD, (B) V_20Gy_, (C) V_10Gy_, and (D) V_5Gy_ among the TBL, NTBLs, and whole lungs. Data are presented as box-and-whisker plots with individual patient data points overlaid. Statistically significant differences (*p* = 0.002) were observed for all parameters between the TBL and the whole-lung, as well as between the TBL and NTBLs, using the Wilcoxon signed-rank test. The TBL-specific dose is markedly higher than the whole-lung average across all metrics. MLD: mean lung dose; TBL: tumor-bearing lobe; NTBLs: non-tumor-bearing lobes; V_20Gy_: relative lung volume receiving at least 20 Gy; V_10Gy_: relative lung volume receiving at least 10 Gy; V_5Gy_: relative lung volume receiving at least 5 Gy

A comprehensive comparison of dosimetric metrics between the whole lungs, TBL, and the combined NTBLs is presented in Figure 3. Statistically significant differences were observed across all evaluated parameters (*p* = 0.002). Specifically, the TBL-specific dose intensities were markedly higher than the conventional whole-lung metrics. The median V_5Gy_, V_10Gy_, and V_20Gy_ for the TBL were 25.7% (IQR, 16.1-27.5%), 12.5% (11.4-15.4%), and 6.3% (5.8-6.8%), respectively, while the corresponding whole-lung values were significantly lower at 8.7% (IQR, 5.6-10.5%), 3.7% (3.0-5.0%), and 1.5% (1.2-2.0%). Similarly, the median MLD within the TBL was 4.61 Gy (IQR, 3.89-4.91 Gy), compared to only 1.68 Gy (IQR, 1.36-2.12 Gy) for the whole lungs. In contrast, the combined NTBLs exhibited minimal radiation exposure, with median V_5Gy_, V_10Gy_, and V_20Gy_ values of 1.6% (IQR, 0.3-7.6%), 0.10% (0.01-2.81%), and 0.03% (0.00-0.69%), respectively, and MLD of 0.65 Gy (IQR, 0.40-1.49 Gy).

Correlation between whole-lung and lobe-specific metrics

**Figure 4:**
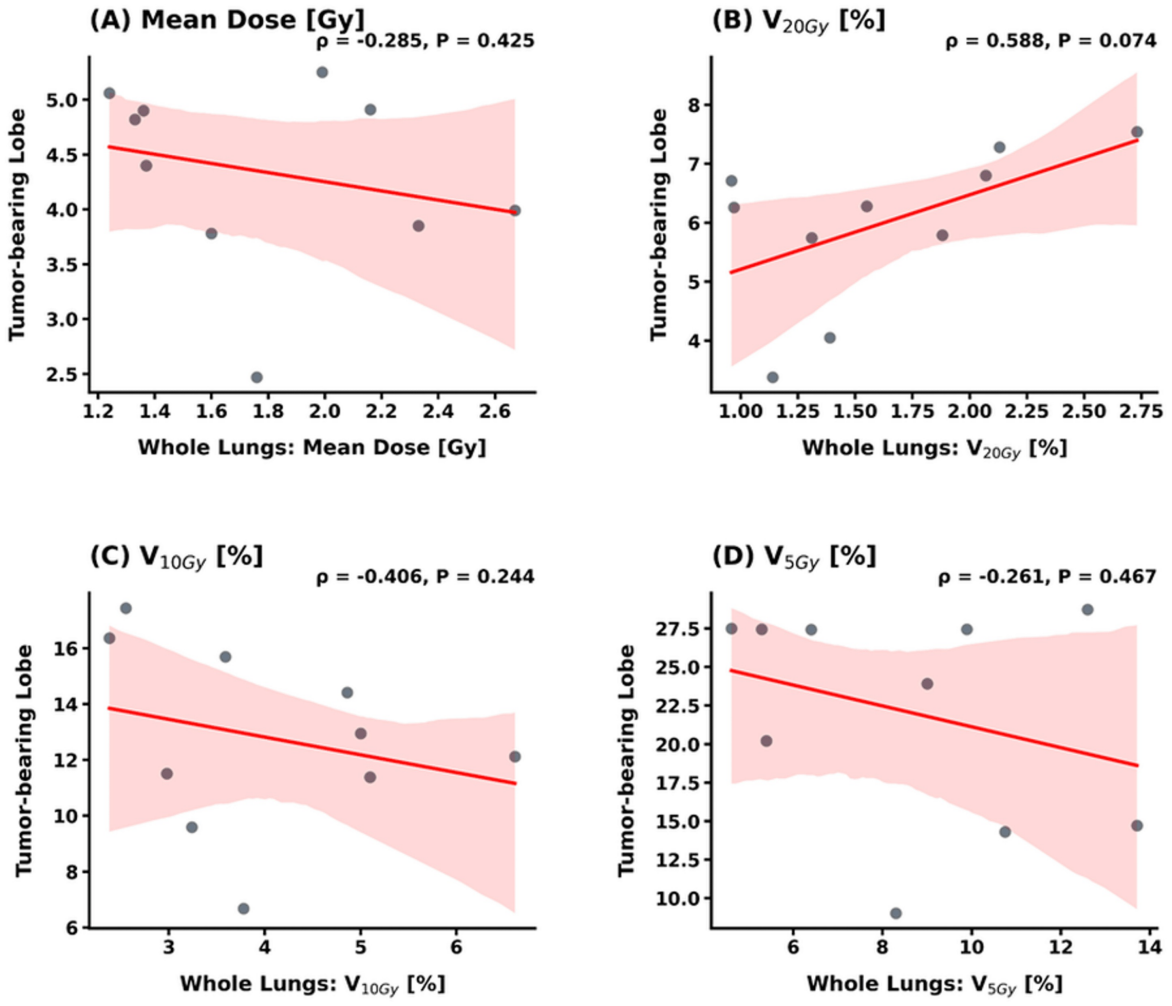
Correlation analysis between whole-lung and tumor-bearing lobe (TBL) metrics Scatter plots with linear regression lines and 95% confidence intervals illustrating the relationship between whole-lung metrics and TBL-specific values for (A) mean dose, (B) V_20Gy_, (C) V_10Gy_, and (D) V_5Gy_. Spearman’s rank correlation coefficient (*ρ*) and *p*-values are indicated for each parameter. While no parameters reached the threshold for statistical significance (*p* < 0.05), a moderate positive trend was observed for V_20Gy_ (*p* = 0.074). V_20Gy_: relative lung volume receiving at least 20 Gy; V_10Gy_: relative lung volume receiving at least 10 Gy; V_5Gy_: relative lung volume receiving at least 5 Gy

**Figure 5:**
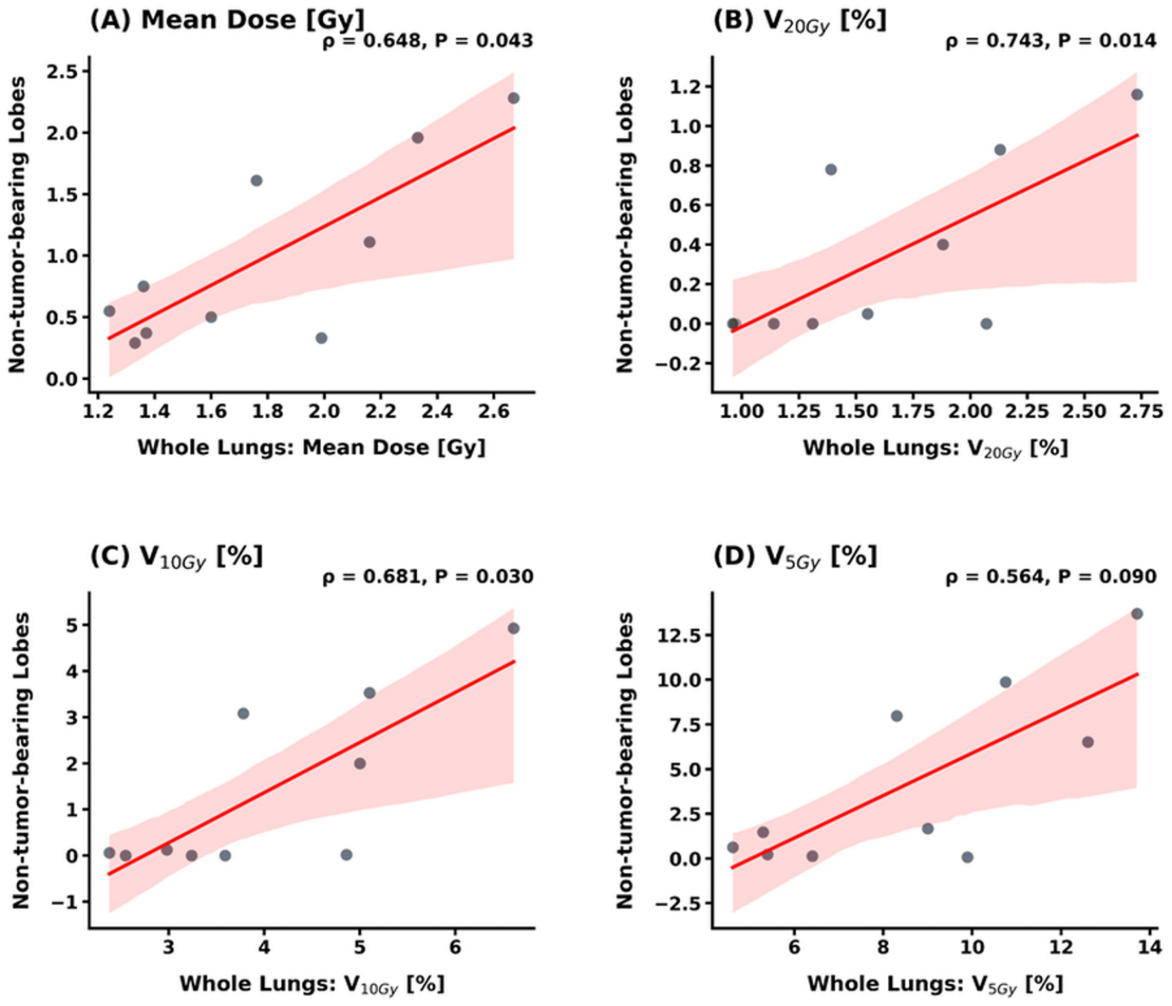
Correlation analysis between whole-lung and non-tumor-bearing lobes (NTBLs) metrics Scatter plots showing the relationship between whole-lung metrics and the dose distributed across the combined NTBLs for (A) mean dose, (B) V_20Gy_, (C) V_10Gy_, and (D) V_5Gy_. In contrast to the TBL analysis, whole-lung metrics exhibit statistically significant positive correlations (*p* < 0.05) with the NTBL dose for MLD, V_20Gy_, and V_10Gy_. MLD: mean lung dose; TBL: tumor-bearing lobe; V_20Gy_: relative lung volume receiving at least 20 Gy; V_10Gy_: relative lung volume receiving at least 10 Gy; V_5Gy_: relative lung volume receiving at least 5 Gy

The correlation between whole-lung metrics and lobe-specific metrics (TBL and NTBLs) is shown in Figures 4-5. For the TBL, no statistically significant correlations were observed with whole-lung metrics for MLD (*ρ* = -0.285, *p* = 0.425), V_10Gy_ (*ρ* = -0.406, *p* = 0.244), and V_5Gy_ (*ρ* = -0.261, *p* = 0.467). Notably, while the correlation for V_20Gy_ also did not reach statistical significance, it showed a moderate positive trend (*ρ* = 0.588, *p* = 0.074) (Figure 4). In contrast, whole-lung metrics exhibited much broader and more consistent correlations with the dose distributed across the combined NTBLs. Specifically, significant positive correlations were observed for MLD (*ρ* = 0.648, *p* = 0.043), V_20Gy_ (*ρ* = 0.743, *p* = 0.014), and V_10Gy_ (*ρ* = 0.681, *p* = 0.030), with V_5Gy_ showing a similar trend (*ρ* = 0.564, *p* = 0.090) (Figure 5).

## Discussion

In this study, we utilized AI-based auto-segmentation software to delineate individual lung lobes and evaluate their lobe-specific dosimetric characteristics in lung SBRT. Quantifying the volumetric distribution of these lobes established the anatomical context for our analysis and defined each lobe's relative contribution to the total lung volume. Previous studies have conducted similar lobar assessments, but those evaluations primarily focused on characterizing volumetric and functional changes during deep inspiration [14,15]. In contrast, our study used images acquired during end-expiratory breath-holding or free breathing, reflecting significant differences in lung volume and density related to different respiratory phases [16].

Our analysis reveals that conventional whole-lung metrics function as a volume-weighted average, which significantly dilutes and obscures the intense radiation concentration localized within the subunit containing the target. V_20Gy_ of the TBL was more than four times higher than that of the whole lungs, while the dose to the NTBLs remained minimal. This systematic discrepancy highlights a clear pattern of dose partitioning that is hidden when evaluating the lung as a single OAR [17]. Furthermore, pulmonary ventilation is not uniformly distributed among lung lobes, with regional differences driven by gravity, compliance, and airway anatomy [18,19]. Physically, high-dose localization is confined within the TBL's anatomical boundaries; however, low-dose spillage transcends these partitions due to technical planning factors, such as the beam arrangement and optimization constraints [20,21].

Our correlation analysis revealed that statistically significant correlations were not reached between whole-lung metrics and the TBL for any evaluated parameters. In particular, the near-threshold *p*-value for V_20Gy_ (*p* = 0.074) suggests that the lack of significance may be due to the limited statistical power of our small cohort rather than a true absence of a relationship. Importantly, while these low-to-mean dose metrics were independent of the target-bearing anatomy, they exhibited strong and consistent correlations with the dose distributed across the combined NTBLs. These findings confirm that conventional whole-lung parameters, particularly MLD and low-dose metrics, primarily function as surrogates for the exposure of uninvolved pulmonary tissue rather than the actual dose delivered to the target lobe. While this study does not report clinical outcomes, this finding is clinically significant because radiation pneumonitis often originates near the high-dose region within the TBL. In SBRT, where high doses are extremely focal, the "volumetric dilution" in whole-lung DVHs may mask high-dose regions that exceed the regenerative capacity of a single lobe. Therefore, quantifying this lobe-specific dose represents a necessary preliminary step toward exploring region-aware toxicity models that explicitly incorporate local dose concentration in future outcome-based studies [22,23].

The emergence of AI-based auto-segmentation software provides a practical means of integrating this organ-specific framework into clinical workflows. These tools offer a more refined dosimetric perspective than traditional whole-lung metrics. However, these findings should be interpreted in light of several limitations. First, since this study did not incorporate clinical outcomes, it cannot establish a direct relationship between lobe-based metrics and the risk of radiation pneumonitis. Second, the study focused on lobe-specific dose-volume metrics and did not evaluate dose distribution quality within each lobe, such as dose conformality. Third, the small sample size (n = 10) limits the statistical power; for instance, the TBL versus whole-lung V_20Gy_ correlation (*ρ* = 0.59, *p* = 0.074) may reflect low power rather than a true absence of correlation. Fourth, the mixture of free-breathing and breath-hold techniques introduces variability in lung volume and density, which could confound DVH comparisons. Finally, the tumor location imbalance (70% in lower lobes) and the use of "combined NTBLs" may mask heterogeneity between individual lobes. These factors necessitate cautious interpretation, and future larger, stratified studies are required to refine these regional dosimetric insights. Despite these limitations, the present study establishes a methodological basis for incorporating lobe-based analysis into the evaluation of lung SBRT.

## Conclusions

This study demonstrated that conventional whole-lung metrics substantially underestimate the radiation dose concentration within the TBL due to volumetric dilution. While high-dose parameters, such as V_20Gy_, reflect local lobar intensity, low-dose spillage behaves as a whole-lung phenomenon, independent of lobar anatomy. Consequently, lobe-based dosimetric analysis provides an anatomically grounded framework that uncovers critical spatial dose distributions otherwise obscured by traditional whole-lung assessment. This approach, facilitated by AI-based auto-segmentation, offers a more refined methodology for evaluating and potentially optimizing lung SBRT treatment plans.
